# Genetic variants in *WDR4* influencing m7G modification are associated with prostate cancer susceptibility

**DOI:** 10.3389/fgene.2026.1867208

**Published:** 2026-10-27

**Authors:** Hesong Jiang, Suqing Diao, Jun Zhao, Xuzhong Liu

**Affiliations:** Department of Urology, The Affiliated Huaian No. 1 People’s Hospital of Nanjing Medical University, Huaian, China

**Keywords:** genetic variants, immunity, m7G, prostate cancer, WDR4

## Abstract

**Introduction:**

The associations between genetic variants in N7-methylguanosine (m7G)-related genes and prostate cancer susceptibility remain unclear. This study investigated the relationship between WDR4 genetic variants and prostate cancer risk and explored their potential regulatory relevance.

**Methods:**

A discovery cohort from the PLCO study and an independent validation cohort from the PRACTICAL consortium were analyzed using logistic regression models. Functional annotation, expression quantitative trait locus analysis, RNA secondary-structure prediction, gene-expression analysis, survival analysis, and single-cell transcriptomic analysis were performed to investigate the potential biological relevance of associated variants.

**Results:**

The rs8130270 variant in WDR4 was associated with prostate cancer susceptibility in the discovery cohort (OR = 0.89, 95% CI = 0.82–0.96, P(FDR) = 2.65 × 10–2) and was validated in the PRACTICAL cohort (OR = 0.98, 95% CI = 0.97–1.00, P = 4.16 × 10–2). The rs8130270 C allele was associated with lower WDR4 expression in prostate tissue. WDR4 expression was higher in prostate cancer tissues, and high WDR4 expression was associated with poorer overall survival (HR = 1.67, 95% CI = 1.06–2.64, P = 2.00 × 10–2). Single-cell analysis showed that WDR4 was expressed across multiple immune and tumor-associated cell populations.

**Discussion:**

These findings suggest that rs8130270 in WDR4 may contribute to prostate cancer susceptibility through effects on WDR4 expression and provide genetic evidence supporting a potential role of WDR4-related m7G regulation in prostate cancer.

## Introduction

Prostate cancer (PCa) is the second most frequently diagnosed malignancy among men globally, accounting for around 1.5 million newly registered cases and 397,000 fatalities annually (Bray et al., 2024). Notably, with over half of newly diagnosed cases occurring in developed regions, prostate cancer poses a substantial global health burden (Seibert et al., 2023). Despite advances in early detection and treatment modalities for prostate cancer, the identification of robust molecular markers for aggressive disease and novel therapeutic targets remains an urgent unmet need (Li et al., 2025). Previous studies have established that both genetic predisposition and environmental factors contribute to prostate cancer pathogenesis (Fontana et al., 2022; Wang et al., 2018). However, identified susceptibility loci account for a fraction of prostate cancer heritability, warranting further investigation into unexplored genetic variants underlying prostate cancer.

N7-methylguanosine (m7G) represents a vital RNA modification with key functions in epigenetic control across both prokaryotes and eukaryotes (Han et al., 2024). This modification affects the generation and maturation of messenger RNA and ribosomal RNA (Yu et al., 2024). Consequently, m7G plays a critical role in diseases, particularly in the progression of cancer. On one hand, the regulatory effects of m7G modifications manifest through multiple mechanisms in tumor biology (Han et al., 2024), and these modifications contribute to fundamental malignant characteristics such as sustained proliferative signaling, evasion of programmed cell death, activation of genomic instability, metabolic reprogramming, invasive and metastatic potential and immune system evasion (Zhang et al., 2024; Chen J. et al., 2024; Lin et al., 2025). On the other hand, abnormal expression profiles of m7G-related molecules are strongly correlated with major clinical and pathological features such as tumor stage, lymph node metastasis, and unfavorable prognosis, indicating their potential to serve as biomarkers for both diagnosis and prognosis in cancer (Fang et al., 2025). Recently, studies have uncovered several key methyltransferase complexes responsible for m7G modifications, including the METTL1/WD repeat domain 4 complex (WDR4), Williams-Beuren syndrome chromosome region 22 (WBSCR22)/methyltransferase activator subunit 11-2 complex (TRMT112), as well as the RNA guanine-7 methyltransferase (RNMT)/RNMT-activating mini-protein (RAM) (Kajal et al., 2026; Du et al., 2023). Among these, the METTL1/WDR4 complex represents one of the most extensively characterized m7G methyltransferase complexes, which comprises METTL1 and its binding partner WDR4, catalyzing the transfer of methyl groups to the N7 position of guanine residues in RNA molecules, modulating RNA functionality (Luo et al., 2022). Additionally, single nucleotide polymorphisms (SNPs) have been shown to influence disease progression and clinical outcomes in various pathological conditions (Tahmin et al., 2025). Notably, polymorphisms in *METTL1* and *WDR4* have been associated with increased Wilms tumor risk (Deng et al., 2023). However, the associations between genetic variants in m7G-related genes and prostate cancer susceptibility remain insufficiently characterized.

The present study aimed to assess the associations between m7G pathway genes and prostate cancer susceptibility using a discovery cohort and a validation cohort. Furthermore, we sought to investigate potential underlying mechanisms that might mediate these genetic associations.

## Materials and methods

### Study subjects

For the discovery phase, a total of 7,654 participants (including 4,581 prostate cancer cases and 3,073 controls, all participants sourced from the United States or Europe), which sourced from the Prostate, Lung, colorectal, and Ovarian (PLCO) Cancer Screening Trial, accessible via the NIH-CDAS repository (https://cdas.cancer.gov/datasets/plco/20/) (Buys et al., 2011). For the validation phase, summary-level data were obtained from the Prostate Cancer Association Group to investigate Cancer Associated Alterations in the Genome (PRACTICAL), which comprised 79,148 prostate cancer cases and 61,106 controls sourced from the Europe (http://practical.ccge.medschl.cam.ac.uk/) (Schumacher et al., 2018) were applied to verify the results of the discovery stage. All studies obtained informed consents from participants, which were approved by relevant institutional ethical committees. The detailed baseline characteristics of the participants have been described in the previous studies.

### Selection of genes and SNPs

Based on previously published reports, seven genes belonging to the m7G pathway were chosen for this study, and the detailed information was presented in the Supplementary Table S1 and Supplementary Figure S1. Differentially expressed genes were identified using The Cancer Genome Atlas (TCGA) database, with the inclusion criteria meeting fold change >1.2 and *P* value <0.05. The STRING database v12.5 (https://string-db.org) was utilized to investigate the protein-protein interaction networks among the candidate genes. In the genetic variant analysis, single-nucleotide polymorphisms (SNPs) located in the target genes were aligned with the European population (EUR) data derived from the 1000 Genomes Project (Phase 3), and the genome assembly version used for SNP coordinates was hg19. Stringent filtering thresholds were applied, which meeting (a) call rate >95%, (b) a minor allele frequency (MAF) ≥ 0.05, and (c) Hardy-Weinberg equilibrium (HWE) ≥ 0.05. Pairwise linkage disequilibrium (LD) analysis was performed to extract tag SNPs, using a threshold of r^2^ ≥ 0.8. Only those SNPs meeting the following criteria were retained: Regulome DB score ≤5, along with evidence of histone marks and motif alterations. This filtering strategy suggests that the retained variants may possess functional relevance. All of the above databases were accessed on 9 October 2025.

### Functional analysis

The RegulomeDB v2.2 (http://RegulomeDB.org/) and HaploReg v4.2 (https://pubs.broadinstitute.org/mammals/haploreg/haploreg.php) websites were utilized to explore the potential functions of candidate SNPs, and enhancer states were predicted using the 3DSNP v2.0 (https://omic.tech/3dsnpv2/). The eQTL analysis was conducted via the Genotype Tissue Expression (GTEx) portal v10 (http://www.gtexportal.org/) to identify target genes that may be regulated by susceptibility-related SNPs, and genes were analyzed using normalized expression, with a *P*-value without adjustment. The secondary structure and minimum free energy (MFE) of RNA were predicted and calculated using the RNAfold web server v2.6.3 (http://rna.tbi.univie.ac.at/cgi-bin/RNAWebSuite/RNAfold.cgi). All of the above websites were accessed on 9 October 2025.

### Gene expression analyses

Differential expression of candidate genes was assessed based on datasets retrieved from The Cancer Genome Atlas (https://cancergenome.nih.gov/), and further validate in the Gene Expression Omnibus repository (https://www.ncbi.nlm.nih.gov/gds/). The impact of expression levels of selected genes on prostate cancer patient survival was further evaluated using the TCGA database. Additionally, gene and protein expression profiles across various tumor tissues and cell types were obtained from TIMER v2.0 (https://cistrome.shinyapps.io/timer/) and the Human Protein Atlas v25.1 (HPA, https://www.proteinatlas.org/). All of the above datasets were accessed on 9 October 2025.

### Single-cell analysis for target genes

Single-cell transcriptome sequencing data from prostate cancer patients were retrieved from the GEO database (accession numbers GSE176031 and GSE143791) to investigate the distribution of *WDR4* expression levels across different immune cell subsets. Quality control, normalization (using SCTransform), dimensionality reduction via Principal Component Analysis (PCA) and Uniform Manifold Approximation and Projection (UMAP), as well as clustering, were carried out with Seurat version 4.3.0.

### Statistical analysis

Unconditional logistic regression was used to evaluate associations between genetic variants and prostate cancer risk under additive, dominant, recessive and codominant genetic models. The models were adjusted for age, body mass index, family history of prostate cancer and the first three genetic principal components. The false discovery rate (FDR) method was conducted to inhibit multiple testing correction. The overall survival time was used as the endpoint for Kaplan-Meier analysis. Patients were divided into high and low *WDR4* expression groups according to the median *WDR4* expression level in the prostate cancer cohort. The hazard ratio (HR), and 95% confidence interval (CI) calculated by multivariate Cox regression analysis with adjustment of age, Gleason score, T-stage, N-stage, M-stage. To compare candidate gene mRNA expression levels derived from TCGA and GEO datasets, Student's t-tests were applied, with statistical significance set at *P* < 0.05.

## Results

### SNPs in *WDR4* and prostate cancer risk

As shown in the flowchart, four out of the seven key genes exhibited differential expression according to the TCGA database, with a fold change >1.2 and *P* < 0.05 in Figure 1. Through a systematic SNP screening process, a total of ten single nucleotide polymorphisms located within four cancer-related genes were eventually obtained for evaluating their associations with prostate cancer risk. The functional annotations of these SNPs are summarized in Supplementary Table S2. In additive genetic models, two SNPs were associated with susceptibility to prostate cancer. After FDR correction for multiple testing, rs8130270 and rs464625 remained statistically significant with prostate cancer (rs8130270: *P*_(FDR)_ = 2.65 × 10^−2^, rs464625: *P*_(FDR)_ = 4.92 × 10^−2^, respectively, Table 1).

**FIGURE 1:**
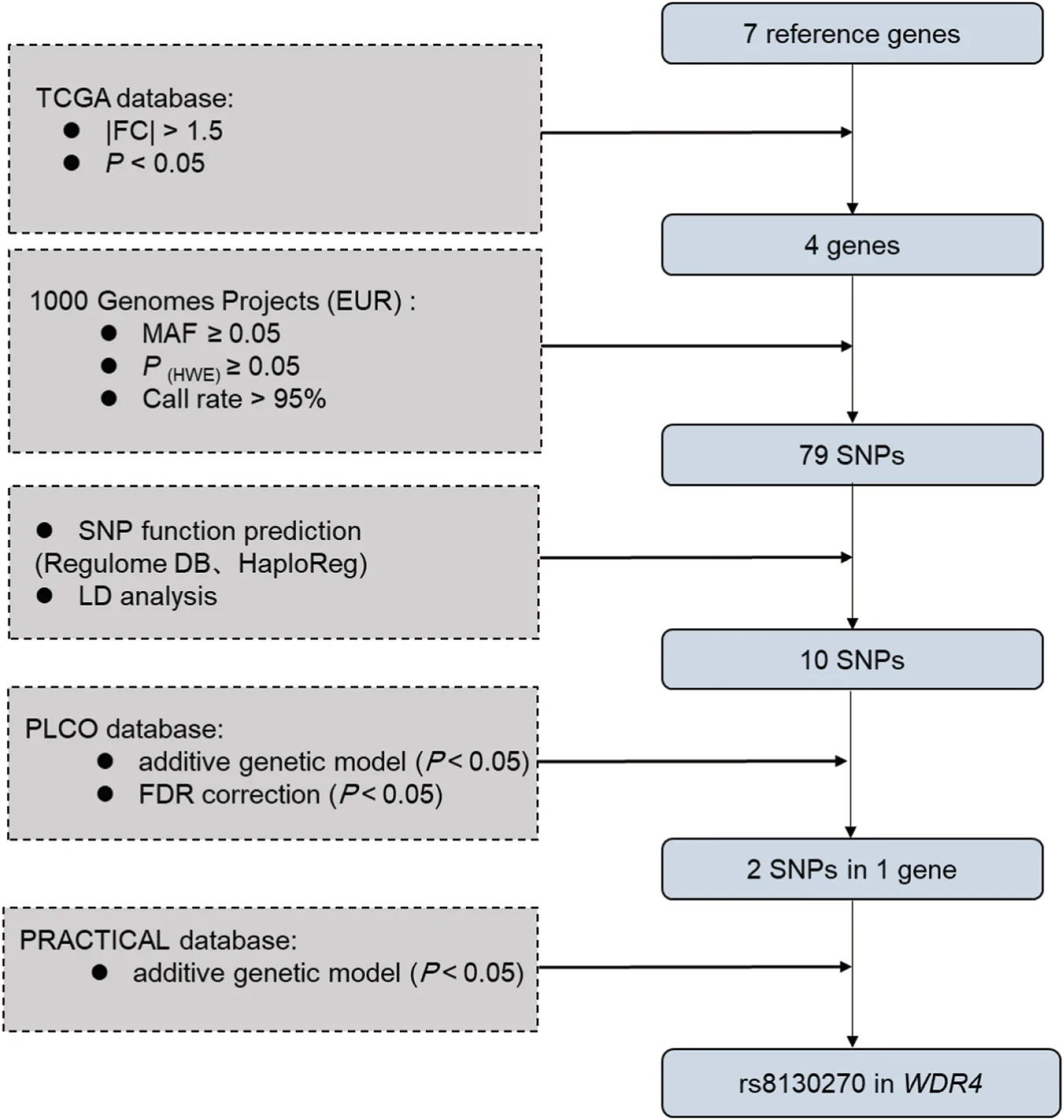
The flow chart of the present study. The workflow of the analysis includes the screening criteria and the methods. Abbreviations: EUR, European population; MAF, minor allele frequency; HWE, Hardy-Weinberg equilibrium; SNP, single nucleotide polymorphism; LD, linkage disequilibrium; FDR, false discovery rate.

**TABLE 1:** Association between SNPs in m7G-associated genes and prostate cancer risk.

Chr	Gene	SNP	Allele^a^	Position	MAF		OR (95% CI)	*P-*value	OR (95% CI)^b^	*P-*value^b^	*P-*value^c^
					Case	Control					
21	*WDR4*	rs8130270	T>C	42860903	0.47	0.49	0.92 (0.86–0.98)	1.12 × 10^−2^	0.89 (0.82–0.96)	2.65 × 10^−3^	2.65 × 10^−2^
21	*WDR4*	rs464625	A>G	42857523	0.32	0.31	1.07 (1.00–1.14)	6.74 × 10^−2^	1.12 (1.03–1.21)	9.84 × 10^−3^	4.92 × 10^−2^
21	*WDR4*	rs4920005	G>A	42856197	0.06	0.05	1.13 (0.99–1.30)	7.94 × 10^−2^	1.12 (0.95–1.32)	1.29 × 10^−1^	4.28 × 10^−1^
21	*WDR4*	rs71320565	G>T	42859345	0.14	0.13	1.07 (0.98–1.18)	1.45 × 10^−1^	1.09 (0.97–1.22)	1.93 × 10^−1^	4.83 × 10^−1^
21	*WDR4*	rs9977828	C>T	42864303	0.42	0.41	1.03 (0.96–1.10)	3.93 × 10^−1^	1.04 (0.97–1.13)	2.54 × 10^−1^	5.08 × 10^−1^
18	*RNMT*	rs13052751	G>A	13759421	0.06	0.07	0.95 (0.84–1.09)	4.76 × 10^−1^	1.05 (0.90–1.23)	2.72 × 10^−1^	4.54 × 10^−1^
21	*WDR4*	rs11911090	A>T	42844358	0.07	0.07	1.04 (0.92–1.19)	5.05 × 10^−1^	1.09 (0.94–1.27)	3.68 × 10^−1^	5.26 × 10^−1^
21	*WDR4*	rs73958033	G>A	42858299	0.06	0.06	1.04 (0.91–1.20)	5.52 × 10^−1^	1.03 (0.88–1.22)	5.09 × 10^−1^	6.37 × 10^−1^
21	*WDR4*	rs71320567	C>T	42863464	0.09	0.09	1.03 (0.92–1.15)	5.94 × 10^−1^	1.06 (0.93–1.21)	7.02 × 10^−1^	7.80 × 10^−1^
18	*RNMT*	rs45561441	G>C	13726683	0.11	0.11	0.98 (0.88–1.09)	6.97 × 10^−1^	0.99 (0.87–1.12)	8.32 × 10^−1^	8.32 × 10^−1^

Chr, chromosome; MAF, minor allele frequency; OR, odds ratio; CI, confidence interval.

^a^ Reference allele > effect allele.

^b^ *P* value of the additive model with adjustment for age, BMI, family history and PC1-3 in the logistic regression model.

^c^ *P* after false discovery rate correction.

Subsequently, the two SNPs were further evaluated in independent validation cohorts. As summarized in Table 2, rs8130270 demonstrated risk effects with prostate cancer in the PRACTICAL database (OR = 0.98, 95% CI = 0.97–1.00, *P* = 4.16 × 10^−2^), and the meta-analysis showed the same trend (OR = 0.98, 95% CI = 0.96–0.99, *P* = 8.79 × 10^−3^).

**TABLE 2:** Association analyses between rs8130270 in *WDR4* and prostate cancer risk in discovery and validation stages.

Stages	Databases	Cases/controls	rs8130270 genotypes (TT/TC/CC)		MAF	*P*	OR (95% CI)^a^	*P* ^a^
			Cases	Controls	Case/control			
Discovery	PLCO	4,581/3,073	1294/2255/1032	787/1546/740	0.47/0.49	1.12 × 10^−2^	0.89 (0.82–0.96)	2.65 × 10^−3^
Validation	PRACTICAL	79,148/61,106	NA	NA	NA	4.16 × 10^−2^	0.98 (0.97–1.00)	4.16 × 10^−2^
​	Combined^†^	83,729/64,179	​	​	​	​	0.98 (0.96–0.99)	8.79 × 10^−3^

OR, odds ratio; CI, confidence interval; NA, not available.

^a^ *P* value of the additive model with adjustment for age, BMI, family history and PC1-3 in the logistic regression model.

^†^ A meta-analysis was performed for combined group.

### Association between rs8130270 and prostate cancer risk

Using four genetic models, we further assessed the association between rs8130270 and prostate cancer risk (Table 3). The frequencies of the TT, TC, and CC genotypes were 28.25%, 49.22%, and 22.53% in cases, and 25.61%, 50.31%, and 24.08% in controls, respectively. Compared with the TT genotype, carriers of the TC/CC genotypes had a significantly reduced risk of prostate cancer (OR = 0.85, 95% CI = 0.75–0.96, *P* = 9.49 × 10^−3^). A significant association was also observed in the recessive model (OR = 0.86, 95% CI = 0.76–0.98, *P* = 2.13 × 10^−2^). These results suggest that the rs8130270 C allele may be associated with a protective effect against prostate cancer.

**TABLE 3:** Association analyses between rs8130270 in *WDR4* and prostate cancer risk.

Genotypes	Cases	Controls	*P*	OR (95% CI)	*P* ^a^	OR (95% CI)^a^
	n (%)	n (%)				
TT	1294 (28.25)	787 (25.61)	​	1.00	​	1.00
TC	2,255 (49.22)	1546 (50.31)	3.23 × 10^−2^	0.89 (0.79–0.99)	5.15 × 10^−2^	0.88 (0.77–1.00)
CC	1032 (22.53)	740 (24.08)	1.27 × 10^−2^	0.85 (0.75–0.97)	2.78 × 10^−3^	0.79 (0.68–0.92)
Additive model TT vs. TC vs. CC			1.12 × 10^−2^	0.92 (0.86–0.98)	2.65 × 10^−3^	0.89 (0.82–0.96)
Dominant model TC + CC vs. TT			1.11 × 10^−2^	0.87 (0.79–0.97)	9.49 × 10^−3^	0.85 (0.75–0.96)
Recessive model CC vs. TT + TC			1.14 × 10^−1^	0.92 (0.82–1.02)	2.13 × 10^−2^	0.86 (0.76–0.98)

OR, odds ratio; CI, confidence interval.

^a^ *P* value with adjustment for age, BMI, family history and PC1-3 in the logistic regression model.

### Stratified analysis of rs8130270 with prostate cancer risk

Stratified analyses were then performed under the dominant genetic model to examine potential associations between rs8130270 and prostate cancer susceptibility across various demographic and clinical subgroups (Supplementary Tables S3–S4). This variant genotype was found to correlate with prostate cancer risk in the subgroups of lymph node (OR = 0.86, 95% CI = 0.76–0.98, *P* = 2.24 × 10^−2^ for N0/N1; OR = 0.82, 95% CI = 0.66–1.02, *P* = 7.67 × 10^−2^ for N2/N3) and metastasis (OR = 0.87, 95% CI = 0.76–0.99, *P* = 2.92 × 10^−2^ for M0; OR = 0.79, 95% CI = 0.63–0.98, *P* = 3.56 × 10^−2^ for M1/M2).

### Potential regulatory function of rs8130270

The annotation details for rs8130270 are provided in the Supplementary Figure S2, and *in silico* analysis suggested the potential functions of the target SNP. As an intronic variant, rs8130270 was predicted to have enhancer activity across two different cell types (Supplementary Table S5). To evaluate the secondary structural changes associated with reference versus effect alleles, the RNAfold web server was employed. The T > C mutation in the rs8130270 was found to alter the RNA secondary structure and modify the minimum free energy (ΔMFE = −1.35 kcal/mol, Figures 2A,B). Furthermore, the eQTL analysis was performed to evaluate whether rs8130270 was associated with *WDR4* expression in the prostate tissue using the GTEx database. The mutant genotype of rs8130270 showed an association with low *WDR4* expression, and the C allele of rs8130270 was associated with reduced prostate cancer risk and lower *WDR4* expression levels (Figure 2C).

**FIGURE 2:**
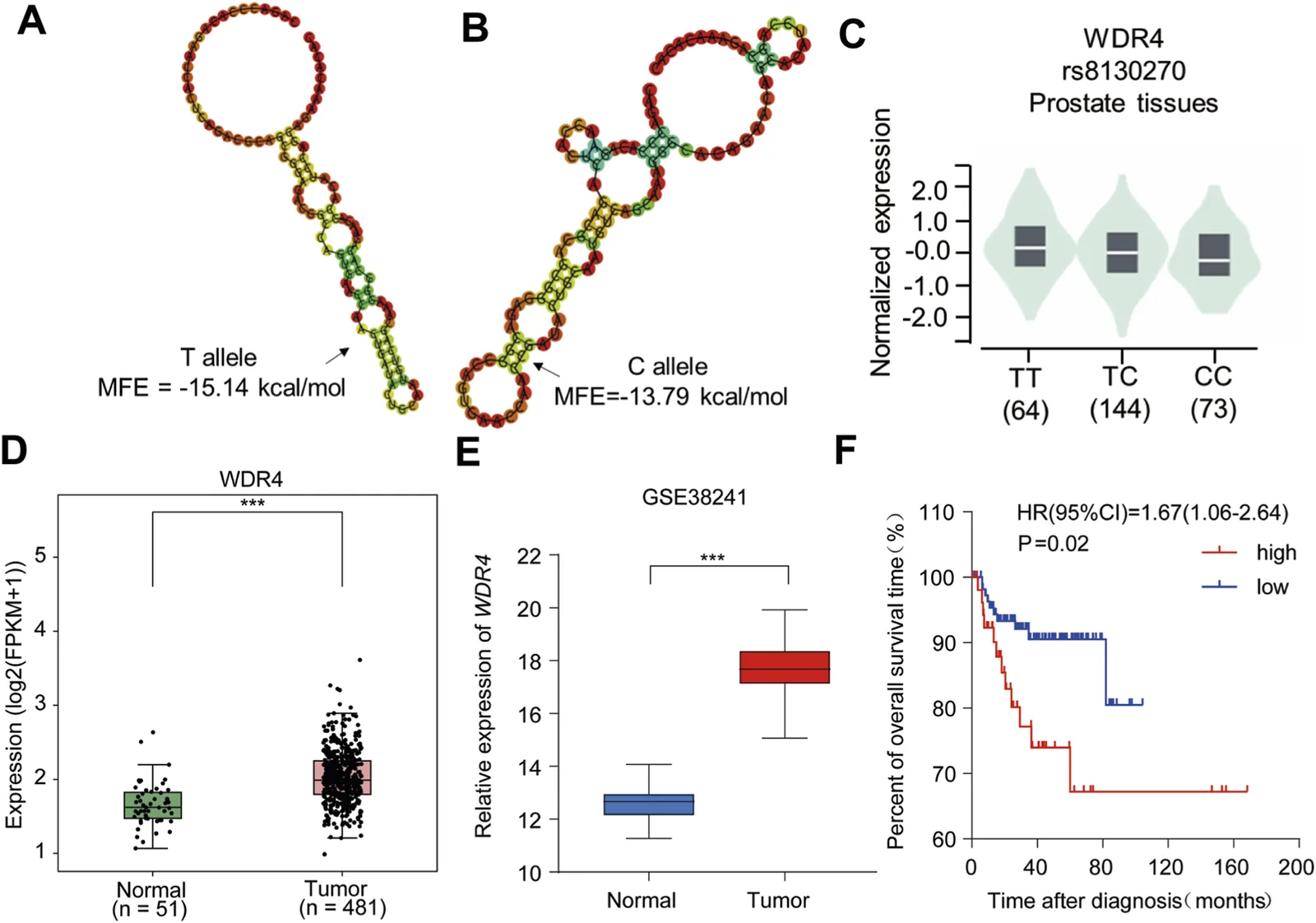
The functional prediction of rs8130270 and analysis of *WDR4* expression and survival. **(A,B)** The prediction of the RNA secondary structure and minimum free energy (MFE) for *WDR4* with the rs8130270 T or C allele (the length of 100 nt centered on rs8130270 was used to predict the secondary structure of RNA, the MFE structure was predicted by a loop-based energy model dynamic programming algorithm, the colors denote consistent/compensatory mutations and the pale colors show pairs absent in some alignments). **(C)** Expression quantitative trait locus (eQTL) analysis of rs8130270 demonstrates the impact of rs8130270 on *WDR4* expression in the prostate tissues using the GTEx dataset, and *P* value without adjustment. *WDR4* levels expressed in prostate tumor tissues and normal tissues in the **(D)** TCGA database and **(E)** GSE38241. **(F)** Kaplan-Meier survival curve of overall survival time based on *WDR4* expression, hazard ratio (HR), and 95% confidence interval (CI) calculated by multivariate Cox regression analysis. *P < 0.05, **P < 0.01, ***P < 0.001.

### Expression levels of *WDR4* in prostate cancer and survival analysis

We evaluated *WDR4* expression in prostate cancer using TCGA and GEO datasets. Compared with adjacent normal tissues, in TCGA data, *WDR4* mRNA levels were significantly elevated in tumor tissues (Figure 2D). This finding was validated using paired tissue analyses from GEO dataset (Figure 2E). *WDR4* expression was also observed in multiple cancer tissues compared with normal tissues (Supplementary Figure S3), and *WDR4* was moderately expressed in prostate tissues across multiple databases (Supplementary Figure S4).

Additionally, Kaplan-Meier analysis was used to evaluate the effect of *WDR4* on prostate cancer survival and revealed that prostate cancer patients with high *WDR4* mRNA levels exhibited poorer survival outcomes (hazard ratio = 1.67, 95% CI: 1.06–2.64, Figure 2F).

### *WDR4* expression at single-cell level

To explore how *WDR4* is distributed across various immune cell populations, we examined single-cell transcriptome sequencing data derived from the GEO database for prostate cancer. Immune cells within the prostate tumor microenvironment were annotated into five major populations in Figure 3A. Notably, *WDR4* expression levels differed between normal and tumor tissues (Figure 3B) using GEO database (GSE176031). Furthermore, using an independent dataset (GSE143791), we observed that *WDR4* was most highly expressed in metastatic tissues (Figures 3D,E). *WDR4* is expressed across different cell populations both these GEO datasets (Figures 3C,F). Collectively, these findings suggest that *WDR4* may associated with the progression of prostate cancer.

**FIGURE 3:**
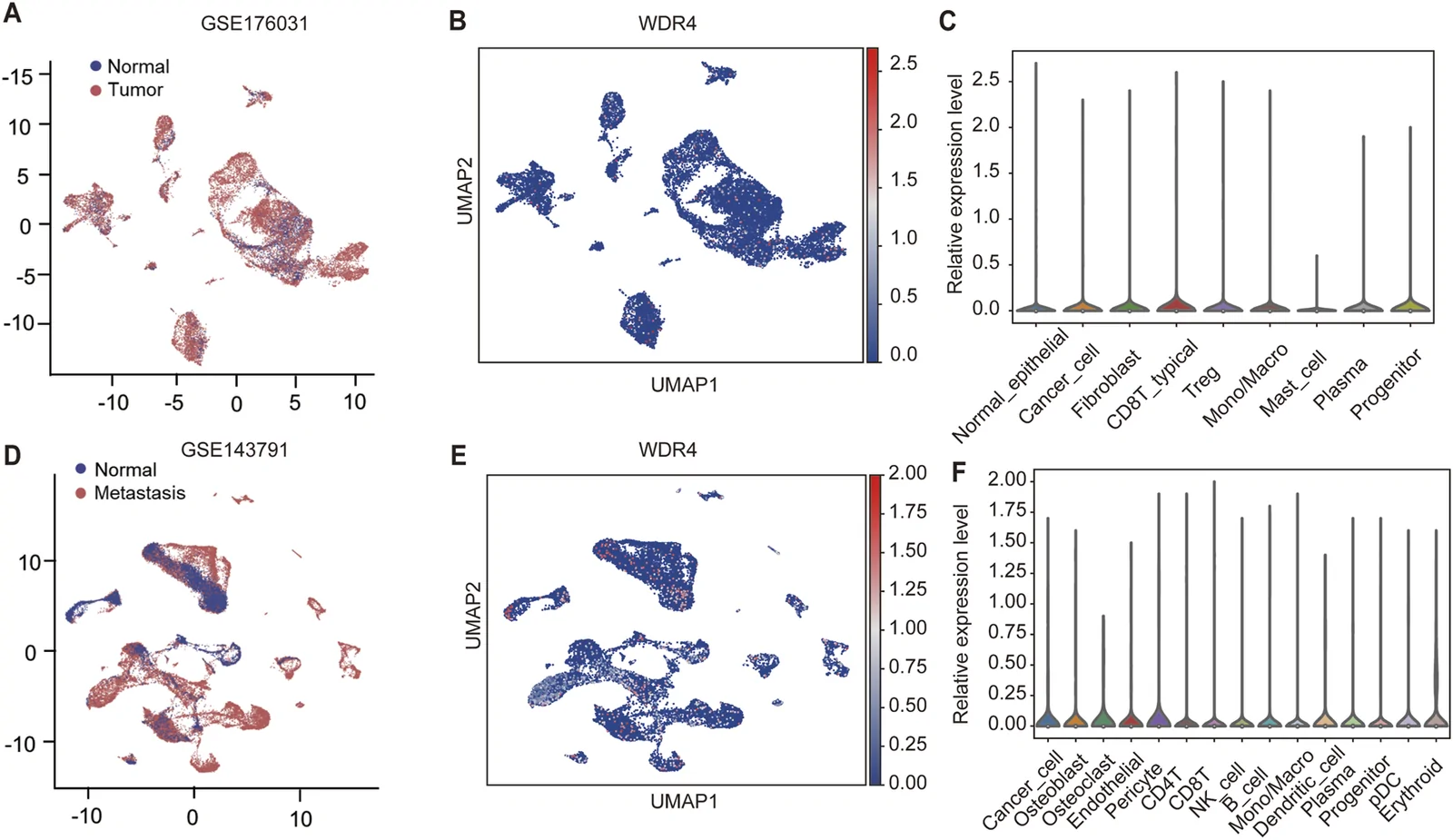
The Single-cell sequencing results for *WDR4*. **(A)** The distribution of tumor tissue and normal tissue cell classes in UMAP plots. **(B)** *WDR4* mRNA expression in different tissues using GSE176031. **(C)** *WDR4* mRNA expression in different cell classes using GSE176031. **(D)** The distribution of metastatic tissue and normal tissue cell classes in UMAP plots. **(E)** *WDR4* mRNA expression in different tissues using GSE143791. **(F)** *WDR4* mRNA expression in different cell classes using GSE143791.

## Discussion

The current study aimed to evaluate the relationship between genetic variants within m7G pathway genes and the risk of developing prostate cancer. During both the discovery and validation phases, rs8130270 in *WDR4* was found to significantly reduce prostate cancer risk. The C allele of rs8130270 was related to low *WDR4* mRNA levels. Additionally, *WDR4* gene expression was enriched in various immune cells. *WDR4* differed in normal and metastatic tissues and prostate cancer patients with high *WDR4* mRNA levels were associated with worse overall survival time. Collectively, these findings imply that *WDR4* has potential relevance to prostate cancer susceptibility.

As one of the most widespread forms of RNA modification, the m7G pathway has recently garnered considerable research interest (Wang et al., 2023). Through influencing the turnover of diverse RNA species—such as messenger RNA, ribosomal RNA, microRNA, and transfer RNA—this modification actively contributes to both physiological and pathological processes (Wu et al., 2025). Increasing evidence highlights the essential role of m7G in the onset and progression of human diseases, particularly cancer (Chen D. et al., 2024). Moreover, abnormal m7G levels are tightly linked to tumor initiation and advancement by modulating the expression of numerous oncogenes and tumor suppressors (Ding et al., 2025). Nevertheless, few investigations have examined the associations between m7G-related genes and prostate cancer risk, and our study identified rs8130270 as a novel factor for prostate cancer risk. In the discovery phase, rs8130270 was associated with a significantly reduced risk of prostate cancer. Moreover, this declining risk was also observed in an independent replication cohort. Nonetheless, heterogeneity was noticed across these populations. Given that participants were recruited from diverse ethnic backgrounds, such heterogeneity might be expected, and a random-effects model was employed for meta-analysis to minimize its impact.

Given that prostate cancer risk is influenced by a variety of factors, we performed stratified analyses across different subgroups. The presence of mutant genotypes was associated with declining prostate cancer risk in cases involving lymph node involvement and distant metastasis. A stronger association was observed between rs8130270 and prostate cancer susceptibility in smoking status compared with nonsmokers. However, these subgroup analyses should be interpreted cautiously because of reduced sample size, multiple comparisons, and potential residual confounding. Therefore, the stratified results should be considered exploratory and require further confirmation in larger independent cohorts.

*WDR4* serves as a subunit of RNA N (7)-methylguanine (m7G) methyltransferase complex (Kajal et al., 2026). Previous study has shown that genetic variants in the *WDR4* gene influence susceptibility to hepatoblastoma in individuals of Chinese Han ethnicity (He et al., 2022). In the current study, *WDR4* rs8130270 was found to reside in an intronic region. Changes in intronic SNPs can influence the secondary structure of precursor mRNA (pre-mRNA), which in turn regulates gene expression by affecting pre-mRNA splicing. Whether rs8130270 affects *WDR4* splicing remains unknown. Interestingly, the T > C mutation was found to alter the RNA secondary structure, and the C allele genotype was associated with lower expression levels of *WDR4* and reduced prostate cancer risk. *WDR4* promotes intrahepatic cholangiocarcinoma progression (Dai et al., 2021), and the same trend was found in prostate cancer. Although the single-cell findings indicate that *WDR4* is expressed in multiple immune and tumor-associated cell populations, they do not establish a direct connection between rs8130270 and cell-type-specific *WDR4* expression. Future studies integrating genotype-resolved single-cell transcriptomic data will be needed to test whether rs8130270 affects *WDR4* expression in specific prostate cell populations.

Certain limitations of the present work need to be recognized. First, dietary habits and other lifestyle variables were not captured in our data collection, and thus could not be incorporated into the stratified analyses accordingly. Second, the exact regulatory mechanisms responsible for our observations have yet to be clarified, and additional molecular biological investigations are necessary. Thirdly, SNP selection was restricted to variants within annotated gene boundaries, and the present study may not capture distal regulatory variants, including enhancers and long-range chromatin-interacting elements. Future fine-mapping, functional studies incorporating promoter, enhancer and chromatin-interaction information are warranted to comprehensively evaluate germline regulation of m7G-related genes in prostate cancer.

In conclusion, our study demonstrated that the *WDR4* genetic variant rs8130270 (T>C) is linked to the risk of prostate cancer. The presence of the rs8130270 C allele influenced the mRNA expression levels of *WDR4*, and higher *WDR4* expression was associated with poorer overall survival time of individuals with prostate cancer. Thus, *WDR4* may play an important role in prostate cancer susceptibility.

## Data Availability

The original contributions presented in the study are included in the article/Supplementary Material, further inquiries can be directed to the corresponding author.
