# Supplementary material for: Genetic variants in WDR4 influencing m7G modification are associated with prostate cancer susceptibility
Source: Front Genet. 2026 Oct 27;17:1867208. doi: 10.3389/fgene.2026.1867208 (PMC13747305; doi:10.3389/fgene.2026.1867208)
Supplement: Supplementary file 1 [file Supplementaryfile1.docx]

***Supplementary Material***

**Genetic variants in *WDR4* influencing m7G modification are associated with prostate cancer susceptibility**

Hesong Jiang^1*^, Suqing Diao^1^, Jun Zhao^1^, Xuzhong Liu^1, †^

^1^ Department of Urology, The Affiliated Huaian No. 1 People’s Hospital of Nanjing Medical University, Huaian, China.

**^†^Correspondence author**

Xuzhong Liu, Ph.D. Department of Urology, The Affiliated Huaian No. 1 People’s Hospital of Nanjing Medical University, Huaian, China; Email: [13915105041@163.com](mailto:13915105041@163.com).

^*^ **First author**

Hesong Jiang，Ph.D. Department of Urology, The Affiliated Huaian No. 1 People’s Hospital of Nanjing Medical University, Huaian, China.

**Supplementary Table 1.** **Differential expression** **analysis of seven key genes in m7G-related genes using TCGA database.**

| Chr | Gene | Position (hg19) | *P* |
| --- | --- | --- | --- |
| 4 | *EIF4E* | 99800427-99850284 | 6.87E-03 |
| 7 | *WBSCR22* | 73097927-73112542 | 6.07E-01 |
| 11 | *TRMT112* | 64083932-64086068 | 4.11E-01 |
| 12 | *METTL1* | 58162254-58165888 | 1.36E-05 |
| 15 | *RAB27A* | 55495164-55582011 | 1.21E-01 |
| 18 | *RNMT* | 13726672-13764555 | 2.32E-04 |
| 21 | *WDR4* | 44263204-44299699 | 2.26E-03 |

**Supplementary Table 2. Functional annotations between the selected 10 SNPs *in silico* analysis.**

| SNPs | Genes | Regulome  DB score | HaploReg v4.1 | | | | | | eQTL *P* value |
| --- | --- | --- | --- | --- | --- | --- | --- | --- | --- |
|  |  |  | Promoter | Enhancer | DNase | Motifs changed | Selected eQTL hits | dbSNP func annot |  |
|  |  |  | histone marks | histone marks |  |  |  |  |  |
| rs8130270 | *WDR4* | 4 | -- | BRN, SPLN | -- | Irf | 19 hits | intronic | 4.50×10^-2^ |
| rs464625 | *WDR4* | 2b | -- | -- | -- | BRCA1,Smad3 | 4 hits | intronic | 3.00×10^-2^ |
| rs4920005 | *WDR4* | 4 | -- | -- | -- | -- | 5 hits | intronic |  |
| rs71320565 | *WDR4* | 5 | -- | ESDR, LNG | BRN | 6 altered motifs | 1 hit | intronic | 6.50×10^-1^ |
| rs13052751 | *WDR4* | 4 | -- | 7 tissues | IPSC,OVRY | Maf | 4 hits | intronic | 3.50×10^-2^ |
| rs73958033 | *RNMT* | 5 | -- | -- | -- | Foxj2 | -- | intronic | 9.30×10^-2^ |
| rs9977828 | *WDR4* | 1f | -- | SKIN, LIV | -- | 10 altered motifs | 16 hits | intronic | 5.40×10^-2^ |
| rs71320567 | *WDR4* | 4 | -- | -- | MUS,MUS | BDP1,PRDM1 | 1 hit | intronic | 7.40×10^-1^ |
| rs11911090 | *WDR4* | 2b | -- | 5 tissues | -- | AP-4,E2A,Myf | 20 hits | synonymous | 2.30×10^-1^ |
| rs45561441 | *RNMT* | 2b | 24 tissues | -- | 53 tissues | 6 altered motifs | 3 hits | 5‘-UTR | 3.90×10^-1^ |

Chr, chromosome.

--, no significant functions were reported for SNPs.

**Supplementary Table 3.** **Stratification analyses of clinicopathologic** variables for association between **rs8130270** and prostate cancer risk.

| Variables | Cases (%) | |  | Controls (%) | | OR (95% CI) | *P* | OR (95% CI)^a^ | *P*^a^ |
| --- | --- | --- | --- | --- | --- | --- | --- | --- | --- |
|  | TC/TT | CC |  | TC/TT | CC |  |  |  |  |
| Age |  |  |  |  |  |  |  |  |  |
| ≥70 | 1466 (71.17) | 594 (28.83) |  | 1901 (74.64) | 646 (25.36) | 0.84 (0.74-0.96) | 8.28×10^-3^ | 0.87 (0.76-0.99) | 3.40×10^-2^ |
| <70 | 1821 (72.23) | 700 (27.77) |  | 385 (73.19) | 141 (26.81) | 0.95 (0.77-1.18) | 6.54×10^-1^ | 0.99 (0.79-1.22) | 8.94×10^-1^ |
| BMI |  |  |  |  |  |  |  |  |  |
| Low | 871 (71.28) | 351 (28.72) |  | 570 (74.61) | 194 (25.39) | 0.84 (0.69-1.04) | 1.06×10^-1^ | 0.73 (0.57-0.93) | 1.20×10^-2^ |
| Middle | 1683 (71.65) | 666 (28.35) |  | 1143 (73.84) | 405 (26.16) | 0.90 (0.77-1.04) | 1.34×10^-1^ | 0.90 (0.76-1.07) | 2.30×10^-1^ |
| High | 678 (71.90) | 265 (28.10) |  | 544 (74.93) | 182 (25.07) | 0.86 (0.69-1.07) | 1.66×10^-1^ | 0.86 (0.66-1.11) | 2.46×10^-1^ |
| Smoking status |  |  |  |  |  |  |  |  |  |
| Never | 1379 (72.50) | 523 (27.50) |  | 856 (72.60) | 323 (27.40) | 0.99 (0.85-1.17) | 9.51×10^-1^ | 0.90 (0.74-1.09) | 2.72×10^-1^ |
| Ever | 1655 (71.34) | 665 (28.66) |  | 1209 (75.19) | 399 (24.81) | 0.82 (0.71-0.95) | 7.63×10^-3^ | 0.85 (0.71-1.01) | 6.24×10^-2^ |
| Current | 253 (70.47) | 106 (29.53) |  | 221 (77.27) | 65 (22.73) | 0.70 (0.49-1.00) | 5.25×10^-2^ | 0.69 (0.45-1.05) | 8.26×10^-2^ |
| Family history |  |  |  |  |  |  |  |  |  |
| Never | 2877 (72.51) | 1091 (27.49) |  | 2080 (74.55) | 710 (25.45) | 0.90 (0.81-1.01) | 6.10×10^-2^ | 0.85 (0.75-0.97) | 1.37×10^-2^ |
| Ever | 347 (67.91) | 164 (32.09) |  | 147 (70.67) | 61 (29.34) | 0.88 (0.62-1.25) | 4.68×10^-1^ | 0.81 (0.53-1.24) | 3.35×10^-1^ |
| Aggressive |  |  |  |  |  |  |  |  |  |
| Never | 1379 (72.50) | 523 (27.50) |  | 856 (72.60) | 323 (27.40) | 0.99 (0.85-1.17) | 9.51×10^-1^ | 0.90 (0.74-1.09) | 2.72×10^-1^ |
| Ever | 1655 (71.34) | 665 (28.66) |  | 1209 (75.19) | 399 (24.81) | 0.82 (0.71-0.95) | 7.63×10^-3^ | 0.85 (0.71-1.01) | 6.24×10^-2^ |

OR, odds ratio; CI, confidence interval.

^a^ *P* value of the dominant model with adjustment for age, BMI, family history and PC1-3 in the logistic regression model.

**Supplementary Table 4.** **Stratification analyses for rs8130270 genotypes and prostate cancer risk.**

| Clinical features | Cases (%) | | OR (95% CI) | *P* | OR (95% CI)^a^ | *P*^a^ |
| --- | --- | --- | --- | --- | --- | --- |
|  | TC/TT | CC |  |  |  |  |
| Gleason score |  |  |  |  |  |  |
| High | 307 (71.56) | 122 (28.44) | 0.87 (0.69-1.09) | 2.11×10^-1^ | 0.86 (0.68-1.09) | 2.21×10^-1^ |
| Middle | 1016 (71.05) | 414 (28.95) | 0.84 (0.73-0.97) | 1.83×10^-2^ | 0.83 (0.70-0.98) | 2.40×10^-2^ |
| Low | 1929 (72.27) | 740 (27.73) | 0.90 (0.80-1.01) | 7.04×10^-2^ | 0.86 (0.75-1.00) | 4.53×10^-2^ |
| Tumor stage |  |  |  |  |  |  |
| I/II | 2863 (71.66) | 1132 (28.34) | 0.87 (0.78-0.97) | 1.07×10^-2^ | 0.85 (0.75-0.96) | 1.06×10^-2^ |
| III/IV | 424 (72.48) | 161 (27.52) | 0.91 (0.74-1.11) | 3.34×10^-1^ | 0.87 (0.68-1.11) | 2.69×10^-1^ |
| N stage |  |  |  |  |  |  |
| N0/N1 | 2884 (71.85) | 1130 (28.15) | 0.88 (0.79-0.98) | 1.70×10^-2^ | 0.86 (0.76-0.98) | 2.24×10^-2^ |
| N2/N3 | 403 (71.08) | 164 (28.92) | 0.85 (0.69-1.03) | 9.91×10^-2^ | 0.82 (0.66-1.02) | 7.67×10^-2^ |
| M stage |  |  |  |  |  |  |
| M0 | 2897 (71.96) | 1129 (28.04) | 0.88 (0.79-0.98) | 2.22×10^-2^ | 0.87 (0.76-0.99) | 2.92×10^-2^ |
| M1/M2 | 390 (70.27) | 165 (29.73) | 0.81 (0.67-0.99) | 4.26×10^-2^ | 0.79 (0.63-0.98) | 3.56×10^-2^ |

OR, odds ratio; CI, confidence interval.

^a^ *P* value of the dominant model with adjustment for age, BMI, family history and PC1-3 in the logistic regression model.

**Supplementary Table 5**. **The effect of rs8130270 *in silico* prediction.**

| SNP | RegulomeDB | |  | 3DSNP v2.0 | |
| --- | --- | --- | --- | --- | --- |
|  | Enhancer histone marks | Motifs changed |  | Enhancer | TFBS |
| rs8130270 | 2 | 2 |  | 2 cell types | 3 |

**Supplementary Figure 1.** **The protein interactions of m7G signaling pathway.** Protein-protein interaction network based on m7G-related genes by STRING database.

**Supplementary Figure 2.** **Functional annotation of rs8130270 in *WDR4* using the 3DSNP database.** The colored tracks represent different regulatory annotation layers, including chromatin state, histone modification, transcription-factor motif alteration and predicted regulatory activity. The labels correspond to genomic features or regulatory elements located near rs8130270. The intertwined lines represent predicted three-dimensional chromatin interactions between the SNP-containing region and potential target regulatory elements or genes.

**Supplementary Figure 3.** **The relative expression levels of *WDR4* expression levels were evaluated in multiple tumor tissue types based on TIMER.**

**Supplementary Figure 4.** Characterization of WDR4 mRNA and protein expression in a variety of tissues. **(A)** FANTOM5 database. **(B)** GTEx database. **(C)** HPA database. **(D)** Consensus database.
